# BH4 Oxidation‐Derived H_2_O_2_ Activates ERK1/2 Signaling via B‐Raf in Rat Dorsal Root Ganglion Neurons

**DOI:** 10.1111/jnc.70271

**Published:** 2025-11-03

**Authors:** Milad Mohammadi, Maike Siobal, Jörg Isensee, Philipp N. Ostermann, Tim Hucho

**Affiliations:** ^1^ Translational Pain Research, Department of Anesthesiology and Intensive Care Medicine, Faculty of Medicine and University Hospital Cologne University of Cologne Cologne Germany

**Keywords:** BH4, B‐Raf, chronic pain, dorsal root ganglion neuron, ERK1/2 signaling, MEK, nociceptor, reactive oxygen species, tetrahydrobiopterin

## Abstract

Elevated Tetrahydrobiopterin (BH4) levels are linked to various pain conditions. Pharmacological or genetic reduction of BH4 levels has analgesic effects in rodent models of neuropathic and inflammatory pain, but also affects neurological and cardiovascular functions. Little is known about the downstream mechanisms of BH4 that could be targeted to attenuate BH4‐induced pain hypersensitivity without lowering BH4 levels. In this study, we exposed ex vivo‐cultured rat dorsal root ganglion (DRG) neurons to BH4 and analyzed the activity of the sensory neuron‐sensitizing kinase ERK1/2 via high‐content imaging. We show that BH4 exposure leads to increased pERK1/2 levels in a dose‐ and time‐dependent manner. Interestingly, we found that H_2_O_2_, as a by‐product of BH4 oxidation and not BH4 itself, induces increased pERK1/2 levels via MEK1/2 and B‐Raf (but not A‐Raf or C‐Raf) and that this can be blocked by pharmacological interference. In conclusion, elevated BH4 levels, as observed in various pain conditions, may drive sensory neuron sensitization via oxidation‐derived H_2_O_2_ and the B‐Raf‐MEK1/2‐ERK1/2 axis, which presents a novel pathway that could be targeted to attenuate BH4‐induced pain hypersensitivity without the necessity to reduce BH4 levels.

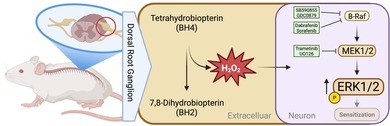

## Graphical abstract

Tetrahydrobiopterin (BH4) is linked to various pain conditions, but targeting its synthesis in vivo is prone to side effects. We now characterized a so far unknown mechanism of BH4‐induced signaling in sensory neurons. In rat dorsal root ganglion (DRG) neurons, we identified neither BH4 nor its oxidation product, BH2, but the byproduct H_2_O_2_ to induce increased pERK1/2 levels via B‐Raf and MEK1/2 in sensory, including nociceptive, neurons. Since ERK1/2 activation via phosphorylation is known to cause sensitization, this H_2_O_2_‐induced B‐Raf‐MEK1/2‐ERK1/2 axis presents a novel pathway that could be targeted to attenuate BH4‐induced pain hypersensitivity.

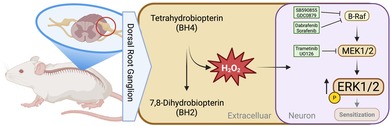

**Abbreviations**
BH2: 7,8‐dihydrobiopterin
BH4: tetrahydrobiopterin
DRG: dorsal root ganglion
ERK1/2: extracellular‐signal regulated kinases
NOS: nitric oxide synthase
PBN: phenyl‐alpha‐tert‐butyl nitrone
RIIβ: RIIβ regulatory subunit of protein kinase A type II
ROS: reactive oxygen species
RRID: Research Resource Identifier (see http://scicrunch.org)

## 1 Introduction

Chronic pain is a global burden, with more than 20% of adults globally reporting long‐lasting or recurrent pain (Breivik et al. 2006; Johannes et al. 2010; Tsang et al. 2008). Effective treatment options remain lacking, as current therapeutics, including nonsteroidal anti‐inflammatory drugs, opioid class therapeutics, and gabapentinoids, do not achieve satisfying pain relief in most chronic pain patients, in part due to therapy‐limiting side effects (Borsook et al. 2014; Woolf 2010; Dib‐Hajj and Waxman 2014). To reduce the individual and socioeconomic burden, there is an urgent need for alternative treatment strategies, especially avoiding adverse side effects (Rice et al. 2016).

Increased tetrahydrobiopterin (BH4) synthesis rates have emerged as an important component in a wide range of pain conditions (reviewed in Cronin et al. 2023; Latremoliere and Costigan 2011; Nasser and Møller 2014). In rodents, nerve damage results in increased BH4 production and its accumulation in dorsal root ganglia (DRG), causing nociceptive hypersensitivity (Tegeder et al. 2006; Latremoliere et al. 2015). Injecting BH4 intrathecally into naïve rats or overexpressing BH4‐producing enzymes in DRG neurons, which include the noxious stimuli‐sensing nociceptors, via genetic approaches, induces pain hypersensitivity (Tegeder et al. 2006; Latremoliere et al. 2015). A recent study demonstrated that, in addition to DRG neurons and macrophages (Latremoliere et al. 2015), mast cells also produce BH4 during skin injury, and that mast cell‐derived BH4 mediates pain hypersensitivity in a mouse model of postoperative pain (Starkl et al. 2024).

In turn, pharmacological or genetic inhibition of key BH4 synthesizing enzymes such as GTP cyclohydrolase 1 (GCH1) and sepiapterin reductase (SRP) has analgesic effects in rodent models of neuropathic and inflammatory pain (Fujita et al. 2020; Tegeder et al. 2006; Latremoliere et al. 2015). Notably, a recent large‐scale phenotypic drug screen identified several compounds that reduce BH4 levels and thus may be used to alleviate chronic pain in the future (Cronin et al. 2022).

However, BH4 is a cofactor in a number of synthesis pathways (e.g., of dopamine, serotonin, and nitric oxide), and reduced BH4 levels are associated with neurological diseases like depression, Alzheimer's, Parkinson's, and cardiovascular diseases (reviewed in Chen et al. 2022; Fanet et al. 2021; Bendall et al. 2014; Latremoliere and Costigan 2011). Thus, analgesic intervention by lowering BH4 levels may be burdened with undesired side effects. To attenuate BH4‐induced pain hypersensitivity without lowering BH4 levels, we must understand the downstream mechanisms that are responsible for BH4‐induced hypersensitivity.

Despite the various studies corroborating an important role of BH4 in pain conditions, the molecular mechanisms are not well explored. So far, exposing ex vivo‐cultured mouse DRG neurons to BH4 has been described to increase intracellular calcium levels (Tegeder et al. 2006; Miyamoto et al. 2009). This was suggested to occur, at least in part, through nitric oxide‐dependent activation of TRPV1 and TRPA1 (Miyamoto et al. 2009). Yet, the authors note that other BH4‐induced pathways may contribute to the observed increase in intracellular calcium, but there is a lack of information regarding such pathways. Moreover, potential calcium‐independent sensitizing mechanisms induced by BH4 have not yet been reported.

Our group uses phosphorylation states of intracellular signaling cascades, such as ERK1/2, as surrogate measurements for the identification of potentially pain‐initiating stimuli (Hucho and Levine 2007; Isensee and Hucho 2019). Monitoring such cascades in thousands of cultured DRG neurons on a single‐cell level using a unique high‐content imaging pipeline, we were able to identify nociception‐modulating extracellular stimuli (Andres et al. 2010, 2013), determine how such stimuli‐induced intracellular signaling modifies analgesic effects and electrical activity (Hucho et al. 2012; Isensee, Krahe, et al. 2017; Yang et al. 2022; Cai et al. 2021), and show that such signaling is, in turn, regulated by electrical activity, thereby altering nociception and pain (Isensee, Schild, et al. 2017; Isensee, Krahe, et al. 2017; van Cann et al. 2021; Isensee et al. 2021; Garza Carbajal et al. 2021).

Using such an analysis of intracellular phosphorylation states of ERK1/2, we now sought to elucidate how elevated BH4 levels result in elevated activity of nociceptors.

## 2 Material and Methods

### 2.1 Animals

Male Sprague Dawley rats (200–225 g, 8–10 weeks old) were obtained from Envigo (now Inotiv, RRID:RGD_737903, Horst, Netherlands). Rats were kept in a temperature‐ and humidity‐controlled animal care facility in IVC‐system cages (Tecniplast, Greenline Double cat. no. GR1800DD) with three to five animals per cage, as recommended by the German Society of Laboratory Animal Science at the University Hospital of Cologne, on a 12‐h light/dark cycle and provided with food and water *ad libitum*. Rats were sacrificed by inhalation of CO_2_ followed by decapitation. Dorsal root ganglia (cervical, lumbar, and thoracic) were removed within 30 min per animal. DRG neurons from different rats were not pooled. DRG neurons from one animal were treated as a single biological replicate. Thus, each data point shown in the graphs corresponds to one animal. All experiments were performed in accordance with the German animal welfare law, with permission from the District Government for Nature and Environment, NRW.

### 2.2 Isolation and Stimulation of Rat DRG Neurons

DRGs were desheathed and incubated in Neurobasal‐A medium (Invitrogen) supplemented with B27 (Life Technologies), L‐Glutamine (Sigma Aldrich), and Glutamate (Sigma Aldrich) and containing collagenase P (Roche; 0.2 U/mL) at 37°C and 5% CO_2_ for 1 h. DRGs were dissociated by trituration with fire‐polished Pasteur pipettes. Axon stumps and cell debris were removed by BSA gradient centrifugation (14% BSA (Sigma), 120× *g*, 8 min). DRG neurons were seeded into 96‐well plates coated with 0.1 mg/mL poly‐L‐ornithine (Sigma Aldrich) and 5 μg/mL laminin (Sigma Aldrich) and incubated overnight (37°C, 5% CO_2_). Neuron density was 1500 neurons/cm^2^. Stimulations were performed by preparing compounds in 10‐fold concentration in 96‐well V‐bottom plates before mixing 50 μL DRG neuron medium with prepared compounds (12.5 μL) and adding these mixtures back to the cells. For stimulations, BH4 (Sigma, cat. no. T4425), NGF (Alomone labs, cat. no. N‐240), OSM (Peprotech, cat. no. 400‐36), ascorbic acid (Sigma, cat. no. A4403), phenyl‐alpha‐tert‐butyl nitrone (PBN) (Enzo, cat. no. ALX‐430‐082‐G001), BH2 (MCE MedChemExpress, cat. no. HY‐W008646), H_2_O_2_ (Applichem, cat. no. A2726,1000), catalase (Sigma, cat. no. C30‐100MG), superoxide dismutase (Sigma, cat. no. S9697‐15KU), 4‐hydroxy‐2,2,6,6‐tetramethylpiperidin‐1‐oxyl (Tempol) (Selleckchem, cat. no. S2910), ML171 (also 2‐APT) (Tocris, cat. no. 4653), AITC (Sigma, cat. no. 377430), capsaicin (Sigma, cat. no. M2028), L‐NAME (Cayman, 51298‐62‐5, cat. no. 80210), L‐NNA (Tocris, cat. no. 0664), L‐NMMA (Tocris, cat. no. 0771), ruthenium red (Abcam, cat. no. ab120264), 2‐Aminoethyl diphenylborinate (2‐APB) (Calbiochem, cat. no. 100065), EGTA (Sigma, cat. no. E4378), BAPTA‐AM (Abcam, cat. no. ab120503), Trametinib (ApexBio, cat. no. A3018), U0126 (Calbiochem, cat. no. 662005), Lifirafenib (Chemietek, cat. no. CT‐BGB283), SB590855 (Axonmedchem, cat. no. Axon 2504), GDC0879 (Selleckchem, cat. no. S110), GW5074 (Selleckchem, cat. no. S2872), ZM336372 (Sigma, cat. no. SML0236), Dabrafenib (Selleckchem, cat. no. S2807), Sorafenib (Tocris, cat. no. 6814), Salirasib (Sigma, cat. no. SML1166), Dasatinib (Sigma, cat. no. CDS023389), Saracatinib (Selleckchem, cat. no. S1006), Src inhibitor 1 (Sigma, cat. no. S2075), Edelfosine (cat. no. 3022), U73122 (Tocris, cat. no. 1268), GO6983 (Peprotech, cat. no. 1331975), Thapsigargin (Calbiochem, cat. no. 586005), Ryanodine (Calbiochem, cat. no. 559276), Dantrolene (Tocris, cat. no. 507), JTV‐519 (Sigma, cat. no. SML0549), Heparin (Tocris, cat. no. 2812), Flufenamic acid (Tocris, cat. no. 4522), N‐(p‐amylcinnamoyl)anthranilic acid (ACAA) (Abcam, cat. no. ab141555), M‐8B (Tocris, cat. no. 5324), STO‐609 (Tocris, cat. no. 1551), KN‐93 (Calbiochem, cat. no. 422708), and Autocamtide‐2‐Related Inhibitory Peptide (AIP) (Calbiochem, cat. no. 189485) were used. All stimulations were performed on day 1 in vitro, i.e., 24 h after trituration and seeding. Cells were fixed 30 min after exposure to BH4 (or OSM and NGF). Pre‐incubations with the described compounds were performed for 10 min unless otherwise stated in the text.

### 2.3 High‐Content Imaging

Rat DRG neurons were fixed at room temperature (RT) with 4% PFA (Roth) and washed twice with PBS before blocking and permeabilizing with 2% normal goat serum (Dianova), 1% BSA, 0.1% Triton‐X‐100 (Roth), and 0.05% Tween 20 (Sigma Aldrich) in PBS for 1 h at RT. DRG neurons were incubated with primary antibodies in 1% BSA in PBS overnight at 4°C, washed three times with PBS, and incubated with secondary antibodies and DAPI (50 ng/mL (Thermo Fisher, cat. no. D1306)) for 1 h at RT in the dark. Primary antibodies were chicken polyclonal anti‐UCHL1 (1:2000, RRID:AB_877619, Novus cat. no. #NB110‐58872), mouse monoclonal anti‐RIIβ (1:2000, RRID:AB_397957, BD Transduction Laboratories cat. no. #610625), and rabbit monoclonal anti‐phospho‐ERK1/2 (1:1000, RRID:AB_2315112, Cell Signaling Technology cat. no. #4370L). Secondary antibodies were highly cross‐adsorbed Alexa Fluor 647– (RRID:AB_162542), 555– (RRID:AB_2535850), and 488– (RRID:AB_2534096) conjugated secondary antibodies (1:1000, Invitrogen). After washing the cells three times with PBS, plates were sealed and scanned with a CX7‐LZR (Thermo Fisher Scientific) HCI system. Images were acquired with a 10× objective and analyzed using the Cellomics software package (Thermo Fisher Scientific). Object selection was based on the criteria: 120–6000 μm^2^; circularity: 1–2; length‐to‐width ratio: 1–2; average intensity: 250–2000; and total intensity: 6 × 10^4^ to 5 × 10^6^. The resulting objects were quantified for average object intensity in the other channels. The UCHL1 channel was used to identify neurons. UCHL1 (previously known as PGP9.5) expression is a well‐established marker to identify sensory neurons among total DRG cells (Genç et al. 2015; Wilson et al. 1988). Only UCHL1‐positive cells, i.e., sensory neurons, were analyzed for pERK1/2 levels in this study. Untreated wells were used for normalization, and compensation was performed to minimize spillover between channels. All analyses were conducted using RStudio as the integrated development environment, as previously described (Isensee and Hucho 2019; Isensee et al. 2021).

### 2.4 Colorimetric Analyses

Colorimetric analysis to determine H_2_O_2_ concentrations has been performed using the Enzo hydrogen peroxide chemiluminescent detection kit (Enzo, cat. no. ADI‐907‐015) following the manufacturer's instructions. Independent standard curves with the H_2_O_2_ standard (0–100 μM H_2_O_2_) were generated for each experimental replicate. Colorimetric analysis of total antioxidant capacity has been performed using the Total Antioxidant Capacity Colorimetric Assay Kit (Elabscience, cat. no. E‐BC‐K219‐M) following the manufacturer's instructions with independent Trolox standard curves (0–1.43 mM Trolox) for each experimental replicate and 1 mM ascorbic acid as a positive control. Spectrophotometric analyses as part of both kits were run on a Multiskan GO Microplate Spectrophotometer (Thermo Fisher Scientific).

### 2.5 Statistical Analysis

Statistical significance between two conditions was tested with unpaired, two‐tailed *t*‐tests, and between more than two conditions by one‐way ANOVA with Bonferroni multiple comparison post hoc testing. Statistical significance between dose–response curves was tested by comparing fitted curves in a nonlinear regression model (three‐parameter, standard Hill slope). The parameters of the model (top, bottom, or the negative logarithm of the EC_50_ values) were compared using the extra‐sum‐of‐squares *F* test. Statistical significance between relative pERK1/2 levels over time between conditions was tested by two‐way ANOVA with Bonferroni's test. Respective *F*‐ and *p*‐values are provided in the figure legends with full statistical reports on multiple comparison tests in Table S1. For all analyses, *p* < 0.05 was considered significant (*p < 0.05). No test for outliers was conducted. Mean values of neurons were considered normally distributed based on previous work using Shapiro–Wilk, Anderson–Darling, or Lilliefors normality tests (Isensee et al. 2018). Replicate experiments were normalized to the mean of multiple unstimulated control wells of each plate to normalize batch variance between experiments performed on different days. This does not lead to data lacking variance. Sample size was determined based on previous studies using our high‐content imaging pipeline using the R package pwr (*α* = 0.05, power = 0.9) (Isensee et al. 2018, 2021; Isensee, Krahe, et al. 2017; Isensee, Schild, et al. 2017).

### 2.6 Software

GraphPad Prism (version 6) has been used for data analysis, including statistical analysis and the generation of data plots. Inkscape (version 1.2) has been used to create figures. BioRender has been used to create schematic illustrations. SkanIt Software 5.0 for Microplate Readers RE (version 5.0.0.42) has been used for spectrophotometric analysis. Microscopic images were taken via the CX7‐LZR platform using studio software version 6.6.2 (Built 8533).

## 3 Results

### 3.1 BH4 Exposure Induces ERK1/2 Activity in Rat Dorsal Root Ganglion Neurons

To explore BH4 downstream mechanisms driving nociceptive hypersensitivity (Latremoliere et al. 2015; Tegeder et al. 2006), we investigated whether exposure of rat primary sensory neurons to BH4 increases ERK1/2 phosphorylation at T202/Y204 (pERK1/2) as a surrogate measurement for ERK1/2 activity and nociceptive sensitization signaling.

**FIGURE 1:**
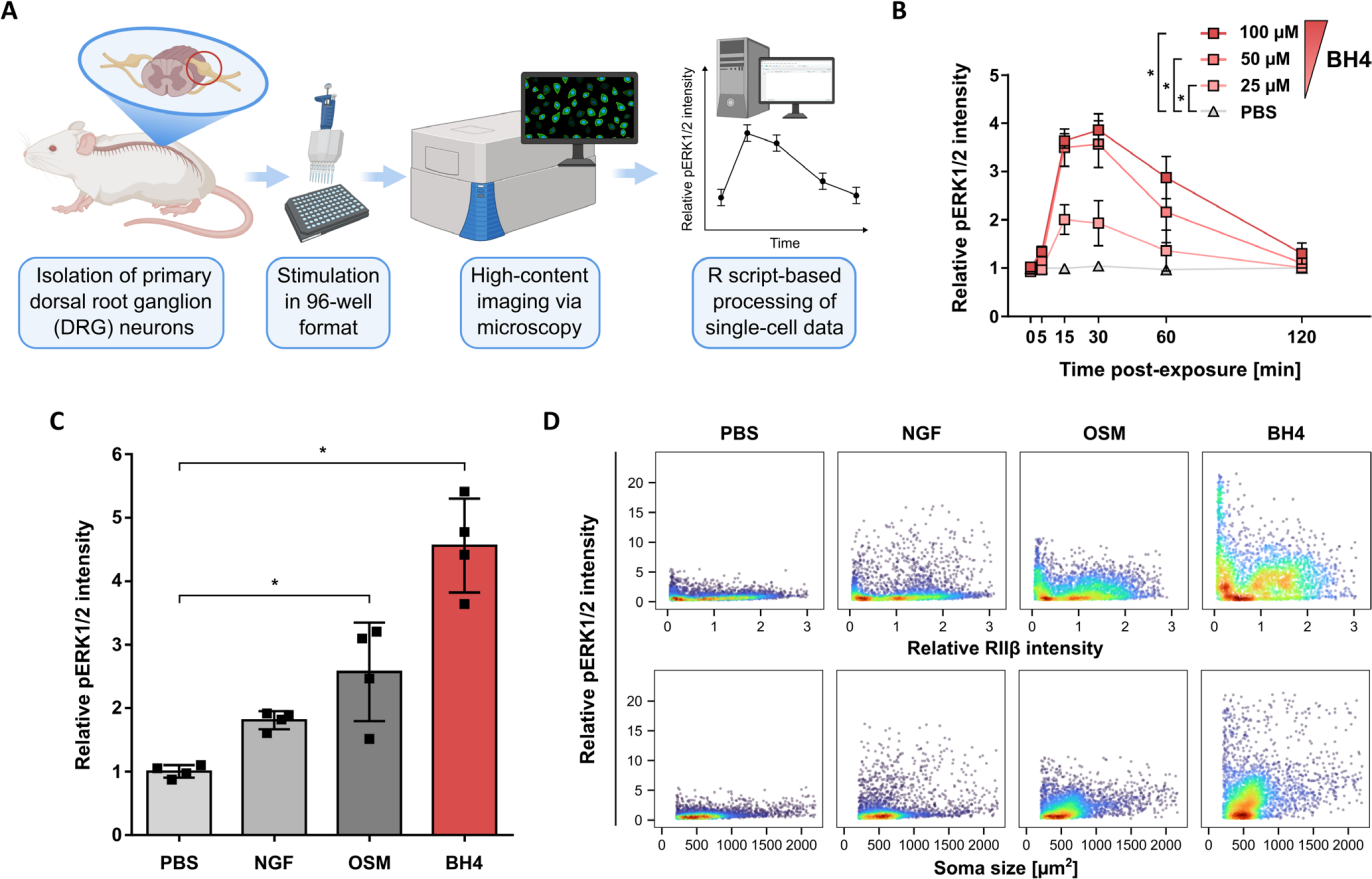
BH4 exposure increases pERK1/2 levels in rat dorsal root ganglion neurons in a time‐ and dose‐dependent manner. (A) Schematic workflow of performed experiments to analyze ERK1/2 activation via determining pERK1/2 levels in ex vivo cultured rat dorsal root ganglion (DRG) neurons by high‐content imaging microscopy and script‐based processing of single‐cell data. (B) Relative pERK1/2 intensity in ex vivo cultured rat DRG neurons (UCHL1^+^) exposed to increasing concentrations of BH4 or PBS for up to 120 min. Statistical significance tested between each concentration and control by two‐way ANOVA with Bonferroni's test (*p < 0.05; 25 μM: *F*(1, 36) = 9.481, *p* = 0.0040; 50 μM: *F*(1, 36) = 51.43, *p* < 0.0001; 100 μM: *F*(1, 36) = 161.8, *p* < 0.0001). Data presented as mean ± SD. (C) Relative pERK1/2 intensity in ex vivo cultured rat DRG neurons (UCHL1^+^) exposed to 100 ng/mL neural growth factor (NGF), 100 ng/mL Oncostatin M (OSM), 100 μM BH4, or PBS as control. Statistical significance tested by one‐way ANOVA with Bonferroni multiple comparison testing between each condition and PBS (*p < 0.05). *F*(3, 12) = 3.028, *p* = 0.0712. Data presented as individual data points representing biological replicates and mean ± SD. (D) Cell density plots showing single cell data of relative pERK1/2 intensity versus relative RIIβ intensity (whole‐cell average set to 1) (upper panel), or versus soma size (lower panel) from neurons shown in (C). (B–D) Experiments have been performed with independent DRG preparations from *n* = 4 animals.

For this, we exposed ex vivo‐cultured DRG neurons from adult rats to increasing concentrations of BH4 for up to 120 min and analyzed pERK1/2 levels in UCHL1‐positive (UCHL1^+^) neurons by high‐content imaging following an established pipeline (Figure 1A) (Isensee and Hucho 2019; Isensee et al. 2018; Isensee, Diskar, et al. 2014; Isensee, Krahe, et al. 2017; Isensee, Schild, et al. 2017; Isensee, Wenzel, et al. 2014). The *UCHL1* (previously known as *PGP9.5*) gene encodes ubiquitin C‐terminal hydrolase L1, and its expression is a well‐established marker to identify sensory neurons among total DRG cells (Genç et al. 2015; Wilson et al. 1988). Our high‐content imaging approach relies on co‐staining for UCHL1 and pERK1/2 at T202/Y204 to selectively analyze pERK1/2 levels in sensory neurons (see Methods). By applying this approach, we observed that BH4 exposure led to a significant dose‐ and time‐dependent increase in intracellular pERK1/2 levels in primary, adult rat DRG sensory neurons (Figure 1B). Since maximum pERK1/2 levels were observed 30 min after BH4 addition (Figure 1B), we selected this timepoint for the analysis of pERK1/2 levels in our following experiments.

Next, we aimed to assess the physiological relevance of BH4‐induced pERK1/2 levels. Therefore, we compared BH4‐induced pERK1/2 levels to pERK1/2 levels after exposure to previously described pain and pERK1/2‐inducing stimuli in rat DRG neurons. We selected nerve growth factor (NGF) (Andres et al. 2012) and oncostatin M (OSM) (Garza Carbajal et al. 2021) at concentrations at which these stimuli induce a robust pERK1/2 response in DRG neurons. As expected, pERK1/2 levels were increased after exposing ex vivo‐cultured rat DRG neurons to NGF or OSM when compared to the control (Figure 1C). Notably, BH4‐induced pERK1/2 levels exceeded pERK1/2 levels after exposure to NGF and OSM by 2.5‐fold and 1.8‐fold, respectively, substantiating that BH4‐induced pERK1/2 levels are relevant for primary sensory neuron sensitization.

Nociceptors can be identified according to high expression of RIIβ, which is a regulatory subunit of protein kinase A type II (Isensee, Diskar, et al. 2014). To assess whether induction of increased pERK1/2 levels by BH4 occurs in nociceptors, we analyzed pERK1/2 levels with respect to RIIβ expression levels. We observed that BH4 induced increased pERK1/2 levels in RIIβ^+^ and RIIβ^−^ DRG neurons, suggesting that BH4 affects all primary sensory neurons, including nociceptors (Figure 1D, upper panel).

Finally, a previous study suggested that the elevation of the BH4 level by overexpressing the BH4‐producing enzyme Gch1 in all sensory neurons using *Advillin–Cre* driver mice mainly affects small‐diameter C‐fiber nociceptors (Latremoliere et al. 2015). To test whether BH4‐induced pERK1/2 levels are limited to small‐diameter neurons, we analyzed pERK1/2 levels with respect to soma size. We found that BH4 increased pERK1/2 levels in small‐diameter neurons and also in large‐diameter neurons (Figure 1D, lower panel).

### 3.2 BH4‐Induced pERK1/2 Levels Are Not Dependent on Nitric Oxide Synthases in Rat DRG Neurons

Next, we set out to elucidate the underlying mechanism of how BH4 exposure leads to increased pERK1/2 levels in rat DRG neurons. BH4 is a cofactor for cellular nitric oxide synthases (NOS) (Latremoliere and Costigan 2011), and BH4 exposure has been described to increase NOS‐derived nitric oxide (Miyamoto et al. 2009). This increase in nitric oxide is believed to modulate TRPA1 and TRPV1 function, resulting in hyperalgesia. Blocking NOS activity has been shown to reduce nocifensive behavior in rodent models of chronic pain (Thomas et al. 1996; Aley et al. 1998; Vizzard et al. 1995; Tegeder et al. 2006; Miyamoto et al. 2009). Importantly, TRPA1 and TRPV1 activation have been shown to induce ERK1/2 activity (Chen et al. 2009; Zhuang et al. 2004; Zhou et al. 2024). Hence, a putative BH4–NOS–TRPA1/TRPV1–ERK1/2 axis would explain the observed BH4‐induced pERK1/2 levels.

To analyze whether a putative BH4–NOS–TRPA1/TRPV1–ERK1/2 axis plays a role, we first sought to confirm that activation of TRPA1 and TRPV1 leads to increased pERK1/2 levels in our setting. For this, we exposed rat DRG neurons to TRPA1 agonist allyl isothiocyanate (AITC) and TRPV1 agonist capsaicin for up to 120 min, and determined pERK1/2 levels. As expected, activation of TRPA1 and TRPV1 induced significantly increased pERK1/2 levels (Figure S1A,B).

**FIGURE 2:**
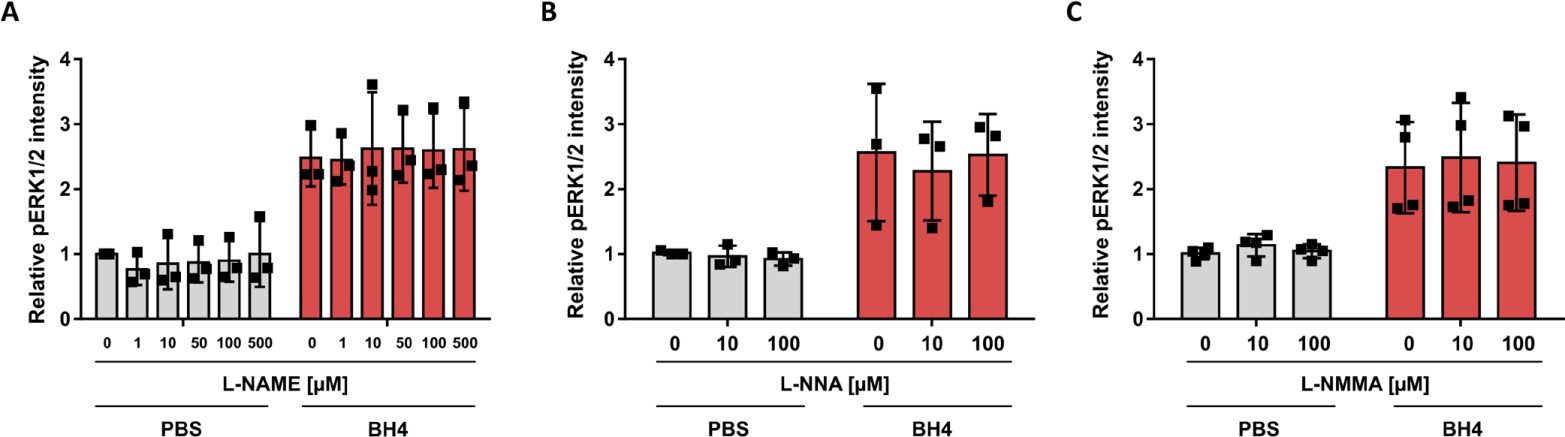
BH4‐induced pERK1/2 levels in rat dorsal root ganglion neurons are not dependent on nitric oxide synthases. (A–C) Relative pERK1/2 intensity in ex vivo cultured rat DRG neurons (UCHL1^+^) exposed to BH4 (100 μM) after treatment with the indicated nitric oxide synthase (NOS) inhibitors. Statistical testing by one‐way ANOVA with Bonferroni multiple comparison testing indicated no significant difference between the 0 μM compound and the increasing compound conditions within the PBS (grey) or BH4 (red) treated groups (*p* > 0.05). Data presented as individual data points representing biological replicates and mean ± SD (A: *F*(5, 12) = 0.05394, *p* = 0.9977; B: *F*(2, 6) = 0.1033, *p* = 9034; C: *F*(2, 9) = 0.04192, *p* = 0.9591). Experiments have been performed with independent DRG preparations from *n* = 3 or 4 animals.

To test whether BH4 exposure may lead to increased pERK1/2 via NOS‐dependent TRPV1 or TRPA1 activation, we pre‐treated rat DRG neurons with the three extensively used competitive NOS inhibitors L‐NAME, L‐NNA, or L‐NMMA (reviewed in Víteček et al. 2012), before the addition of BH4. Inhibition of NOS activity with increasing concentrations of up to 500 μM L‐NAME and 100 μM of L‐NNA and L‐NMMA did not reduce BH4‐induced pERK1/2 levels (Figure 2A–C). Importantly, these NOS inhibitors exhibit inhibitor affinity constants (*K*_*i*_) in the low micromolar range of below 3 μM for all NOS isoforms (Víteček et al. 2012), and L‐NAME has been shown to inhibit intracellular calcium increase in mouse DRG neurons in response to 1 mM BH4 at a concentration of 200 μM. Therefore, we conclude that the observed BH4‐induced increase in pERK1/2 levels in rat DRG neurons is most likely not dependent on cellular NOS.

### 3.3 BH4 Oxidation‐Derived H_2_O_2_ and Not BH4 Itself or BH2 is Responsible for Increased pERK1/2 Levels in Rat DRG Neurons

**FIGURE 3:**
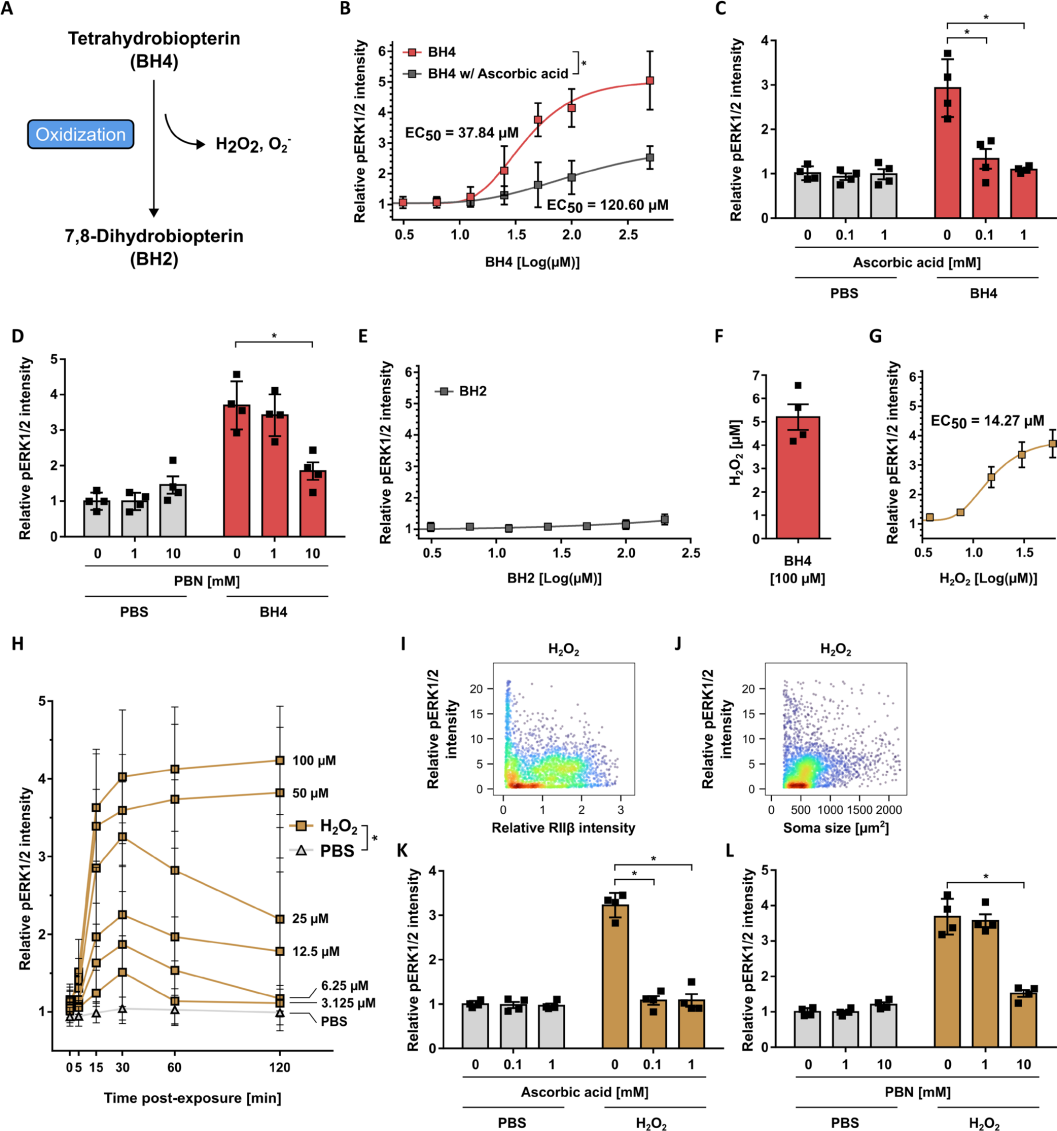
Oxidation‐derived reactive oxygen species like H_2_O_2_ and not BH4 itself or BH2 drive ERK1/2 signaling in rat dorsal root ganglion neurons following treatment with BH4. (A) Schematic illustrating the generation of 7,8‐dihydrobiopterin (BH2) and H_2_O_2_ by BH4 oxidation. (B) Dose–response curve of relative pERK1/2 intensity in ex vivo cultured rat DRG neurons (UCHL1^+^) exposed to BH4 with or without ascorbic acid (100 μM). Statistical significance tested by comparing fitted curves in a nonlinear regression model (three‐parameter, standard Hill slope). Data presented as mean ± SD. *F*(3, 76) = 57.83, *p* < 0.0001 (C, D) Relative pERK1/2 intensity in ex vivo cultured rat DRG neurons (UCHL1^+^) exposed to BH4 (100 μM) after treatment with the indicated antioxidizing compounds. (E) Dose–response curve of relative pERK1/2 intensity in ex vivo cultured rat DRG neurons (UCHL1^+^) exposed to BH2. Data presented as mean ± SD. (F) Colorimetric analysis of H_2_O_2_ levels generated by 100 μM BH4 in PBS using the *Enzo* hydrogen peroxide chemiluminescent detection kit following manufacturer's instructions with independent H_2_O_2_ standard curves (0–100 μM H_2_O_2_) for each replicate. Data presented as individual data points representing biological replicates and mean ± SD. (G) Dose–response curve of relative pERK1/2 intensity in ex vivo cultured rat DRG neurons (UCHL1^+^) exposed to H_2_O_2_. Data presented as mean ± SD, and curve‐fitted line in a nonlinear regression model (three‐parameter, standard Hill slope). (H) Relative pERK1/2 intensity in ex vivo cultured rat DRG neurons (UCHL1^+^) exposed to increasing concentrations of H_2_O_2_ or PBS as a control for up to 120 min. Statistical significance tested between H_2_O_2_ exposure and PBS control by two‐way ANOVA with Bonferroni's test (*p < 0.05; *F*(6, 125) = 39.22, *p* < 0.0001). Data presented as mean ± SD. (I, J) Cell density plots showing single cell data of relative pERK1/2 intensity versus relative RIIβ intensity (whole‐cell average set to 1) (I), or versus soma size (J) from neurons exposed to 25 μM H_2_O_2_. (K, L) Relative pERK1/2 intensity in ex vivo cultured rat DRG neurons (UCHL1^+^) exposed to H_2_O_2_ (25 μM) or PBS after treatment with the indicated antioxidizing compounds. (C, D, K, L) Statistical significance tested by one‐way ANOVA with Bonferroni multiple comparison testing, in comparison to 0 μM compound (*p < 0.05). Data presented as individual data points representing biological replicates and mean ± SD. (C: *F*(2, 9) = 19.03, *p* = 0.0006; D: *F*(2, 9) = 11.41, *p* = 0.0034; K: *F*(2, 9) = 95.40, *p* < 0.0001; L: *F*(2, 9) = 42.13, p < 0.0001). (B–E, G–L) Experiments have been performed with independent DRG preparations from *n* = 4 animals.

Beyond the described effect on cellular NOS (Miyamoto et al. 2009), no other downstream BH4‐dependent mechanism in DRG neurons has been reported so far. Notably, BH4 is prone to rapid oxidation in an aqueous environment, which is sometimes neglected in experimental studies (Heller et al. 2001). Oxidation of BH4 results in 7,8‐dihydrobiopterin (BH2), a reaction well described to generate reactive oxygen species (ROS) (Figure 3A) (Garry et al. 2009; Kirsch et al. 2003). Therefore, we now sought to assess whether BH4 or any (by)‐products of its oxidation are responsible for the observed increase in pERK1/2 levels in rat DRG neurons.

We first investigated whether BH4 is the molecule that itself induces the elevation of pERK1/2 levels. We used ascorbic acid, which is a water‐soluble antioxidant that chemically stabilizes BH4 and decreases the process of oxidation at the selected concentrations of up to 100 μM (Baker et al. 2001; Heller et al. 2001). Supplementing the BH4‐containing medium with 100 μM ascorbic acid significantly decreased the BH4‐induced ERK1/2 activity in rat DRG neurons (Figure 3B).

To exclude that ascorbic acid alone reduces pERK1/2 levels in rat DRG neurons independent of its action on BH4, we analyzed pERK1/2 levels after exposing the neurons to ascorbic acid in the absence of BH4. Ascorbic acid did not change pERK1/2 levels in rat DRG neurons at 0.1 and 1 mM (Figure 3C). Thus, it is most likely ascorbic acid's effect on BH4 that prevents increased pERK1/2 levels upon BH4 exposure. Moreover, we observed during the same experiment that increasing the concentration of ascorbic acid to 1 mM completely abolished BH4‐induced ERK1/2 activity (Figure 3C). To further confirm that this reduction is due to the antioxidizing capacity of ascorbic acid and not due to other properties of this chemical, we repeated this experiment with a chemically unrelated antioxidant, phenyl‐alpha‐tert‐butyl nitrone (PBN). Again, we observed a significant reduction in pERK1/2 levels, suggesting that not BH4 itself, but rather a product of its oxidation is responsible for the observed induction of elevated pERK1/2 levels (Figure 3D).

The prominent oxidation product of BH4 is BH2. Thus, we next analyzed whether exposure to BH2 induces increased pERK1/2 levels. We exposed rat DRG neurons to increasing concentrations of BH2. However, we did not observe an increase in pERK1/2 levels, indicating that BH2 is not responsible for increasing pERK1/2 levels upon BH4 exposure (Figure 3E).

Therefore, we assumed that ROS, generated as a by‐product of BH4 oxidation, are responsible. Previous studies have demonstrated that BH4 oxidation generates hydrogen peroxide (H_2_O_2_) and superoxide anions (O_2_^−^) (Kirsch et al. 2003; Davis and Kaufman 1993). Interestingly, H_2_O_2_ is increasingly recognized as a second messenger (reviewed in Ray et al. 2012; Forman et al. 2010), and H_2_O_2_ exposure has been shown to induce ERK1/2 activation in neural stem cells (Ruan et al. 2024) and nonneural cells (Abe et al. 1998; Nishida et al. 2000).

To investigate whether H_2_O_2_ drives the observed BH4‐induced increase in pERK1/2 levels in rat DRG neurons, we first assessed the H₂O₂ production in our setting by testing whether the applied BH4 concentration of 100 μM generates detectable H_2_O_2_ levels using a colorimetric assay. Based on H_2_O_2_ standards with concentrations ranging from 0 μM to 100 μM (data not shown), and in agreement with others (Kirsch et al. 2003), we found that 100 μM BH4 produces 5.2 ± 1 μM H_2_O_2_ (Figure 3F). Subsequently, we analyzed whether direct exposure to H_2_O_2_ elevates pERK1/2 levels in rat DRG neurons. H_2_O_2_ dose‐dependently elevated pERK1/2 levels with an EC_50_ value of 14.27 μM (Figure 3G). Moreover, a time‐course experiment with different H_2_O_2_ concentrations substantiated that BH4 oxidation‐derived H_2_O_2_ is sufficient to elevate pERK1/2 levels in rat DRG neurons (Figure 3H).

To corroborate our hypothesis that BH4‐derived H_2_O_2_ is responsible for the observed increased pERK1/2 levels, we analyzed whether H_2_O_2_ increased pERK1/2 levels occur in the same primary sensory neurons when compared to BH4. Thus, we studied pERK1/2 levels upon H_2_O_2_ exposure with respect to soma size and RIIβ expression level with single‐cell resolution, as performed after exposure to BH4. This analysis showed that elevated pERK1/2 levels upon H_2_O_2_ exposure were not limited to neurons of a certain soma size and that RIIβ^+^ as well as RIIβ^−^ neurons respond with elevated pERK1/2 levels (Figure 3I,J). Hence, both single‐cell analyses provided the same results when compared to the analyses after exposure to BH4 (Figure 1D,E).

In addition to this single‐cell‐based comparison, we supplemented the H_2_O_2_‐containing medium with antioxidants ascorbic acid and PBN to test whether it is the oxidizing capacity of H_2_O_2_ that drives elevation of pERK1/2 levels in rat DRG neurons. Adding these two antioxidants significantly reduced H_2_O_2_'s effect on pERK1/2 levels, indicating that BH4 and H_2_O_2_ induce ERK1/2 activity via the same mechanism (Figure 3K,L). Overall, these experiments show that BH4 oxidation‐derived ROS, such as H_2_O_2_ and not BH4 itself or BH2, drive ERK1/2 signaling in rat DRG neurons.

**FIGURE 4:**
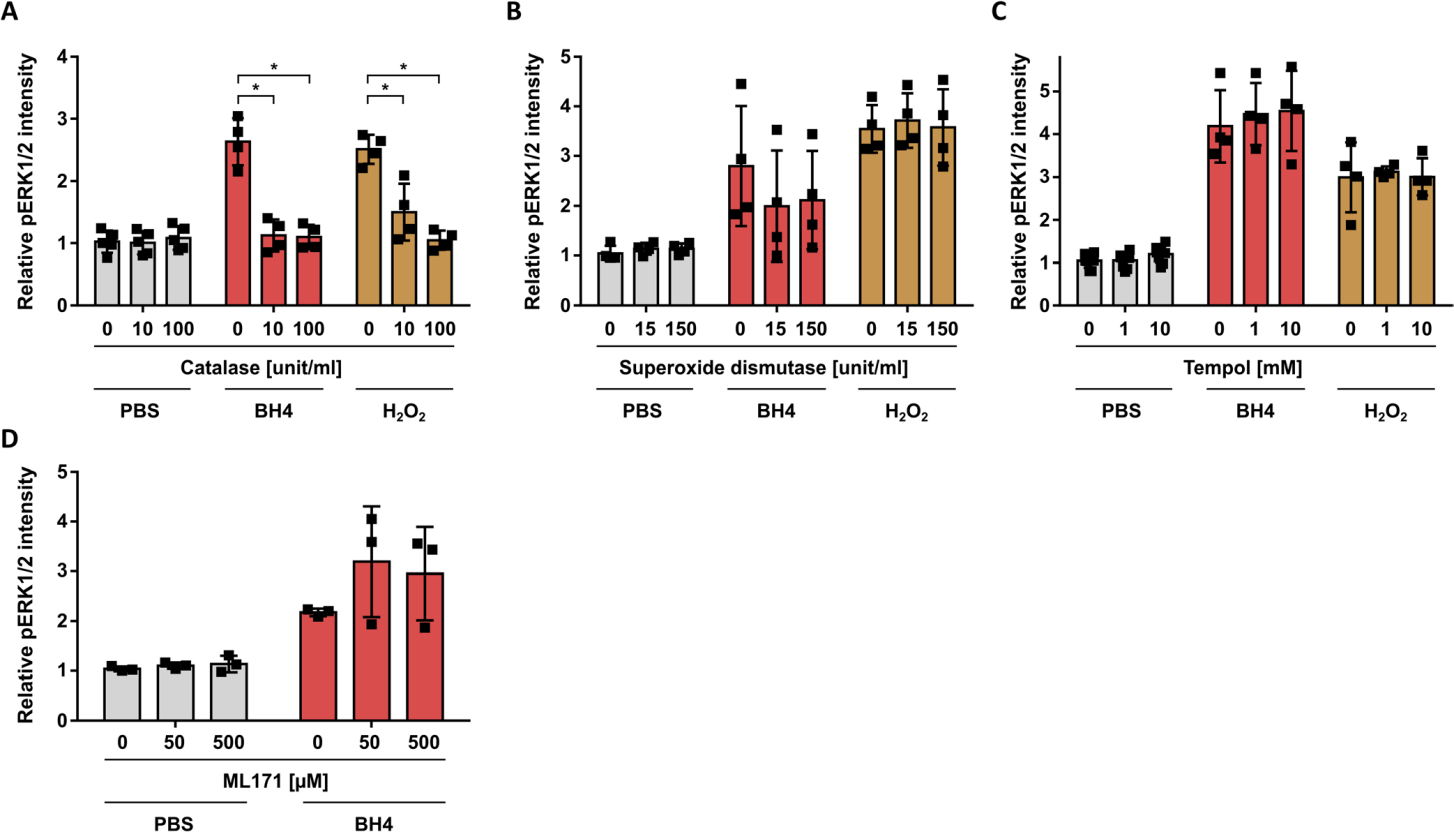
Oxidation‐derived H_2_O_2_ drives BH4‐induced ERK1/2 signaling in rat dorsal root ganglion neurons. (A–D) Relative pERK1/2 intensity in ex vivo cultured rat DRG neurons (UCHL1^+^) exposed to BH4 (100 μM) or H_2_O_2_ (25 μM) after treatment with the indicated antioxidizing or blocking compounds. Statistical significance tested by one‐way ANOVA with Bonferroni multiple comparison testing, in comparison to 0 μM compound (*p < 0.05). Data presented as individual data points representing biological replicates and mean ± SD. (A: BH4, *F*(2, 9) = 37.25, *p* < 0.0001; H_2_O_2_, *F*(2, 9) = 23.39, *p* = 0.0003). Experiments have been performed with independent DRG preparations from *n* = 4 animals.

Since previous studies showed generation of H_2_O_2_ and O_2_^−^ through BH4 oxidation, but mostly H_2_O_2_ is proposed as a second messenger (Forman et al. 2010), we next sought to determine which ROS contributes the most to BH4‐induced pERK1/2 levels. To first confirm that specifically H_2_O_2_ has the capacity to induce ERK1/2 phosphorylation, we pre‐treated cells with catalase before exposure to BH4 or H_2_O_2_. Catalase rapidly breaks down H_2_O_2_ into water and oxygen. Thus, inhibition of BH4‐induced ERK1/2 activity by catalase would strongly support a role of H_2_O_2_. We observed that catalase completely prevented the increase in pERK1/2 levels induced by BH4 or H_2_O_2_ (Figure 4A). Nevertheless, to exclude the possibility that the more reactive O_2_^−^ ROS contributes to BH4's effect on pERK1/2 levels, we also used superoxide dismutase and Tempol to scavenge O_2_^−^. However, neither superoxide dismutase nor Tempol reduced the BH4‐ or H_2_O_2_‐induced pERK1/2 levels (Figure 4B,C). Based on these findings, we conclude that BH4 oxidation‐derived H_2_O_2_ is the major driver of ERK1/2 signaling in rat DRG neurons upon BH4 exposure.

Finally, the enzyme Nox1 (NADPH oxidase 1) has been shown to produce ROS, and this has been suggested to play a role in sensitizing sensory neurons (Kumar et al. 2019). Blocking Nox1 activity with the specific inhibitor ML171 reduced nocifensive behavior in a mouse model of formalin‐induced nociception (Kumar et al. 2019). To test whether Nox1‐dependent ROS production contributes to the here observed BH4‐induced pERK1/2 levels, we applied ML171 to our rat DRG neurons and determined pERK1/2 levels following BH4 treatment. We did not observe any effect of the specific Nox1 inhibitor ML171 on BH4‐induced pERK1/2 levels (Figure 4D). This suggests that Nox1 activity does not play a role in BH4‐induced ERK signaling in rat DRG neurons.

### 3.4 Ca^2+^ Signaling is Likely Not Involved in BH4‐ and H_2_O_2_ ‐Induced pERK1/2 Levels

It has been reported that BH4 exposure induces Ca^2+^ influx into DRG neurons (Tegeder et al. 2006). H_2_O_2_ is likewise associated with Ca^2+^ influx into primary sensory neurons (Mohammadi et al. 2018; Oehler et al. 2020). Ca^2+^ is a ubiquitous intracellular signaling messenger that is involved in different pathways leading to ERK1/2 activity (Schmitt et al. 2004). To investigate whether Ca^2+^ influx, i.e., Ca^2+^‐dependent signaling, is of relevance or BH4‐ and H_2_O_2_‐induced increased pERK1/2 levels, we treated rat DRG neurons with different chemicals and reagents to interfere with Ca^2+^ signaling, starting with broad‐spectrum approaches while stimulating the cells with BH4 or H_2_O_2_ in the following.

**FIGURE 5:**
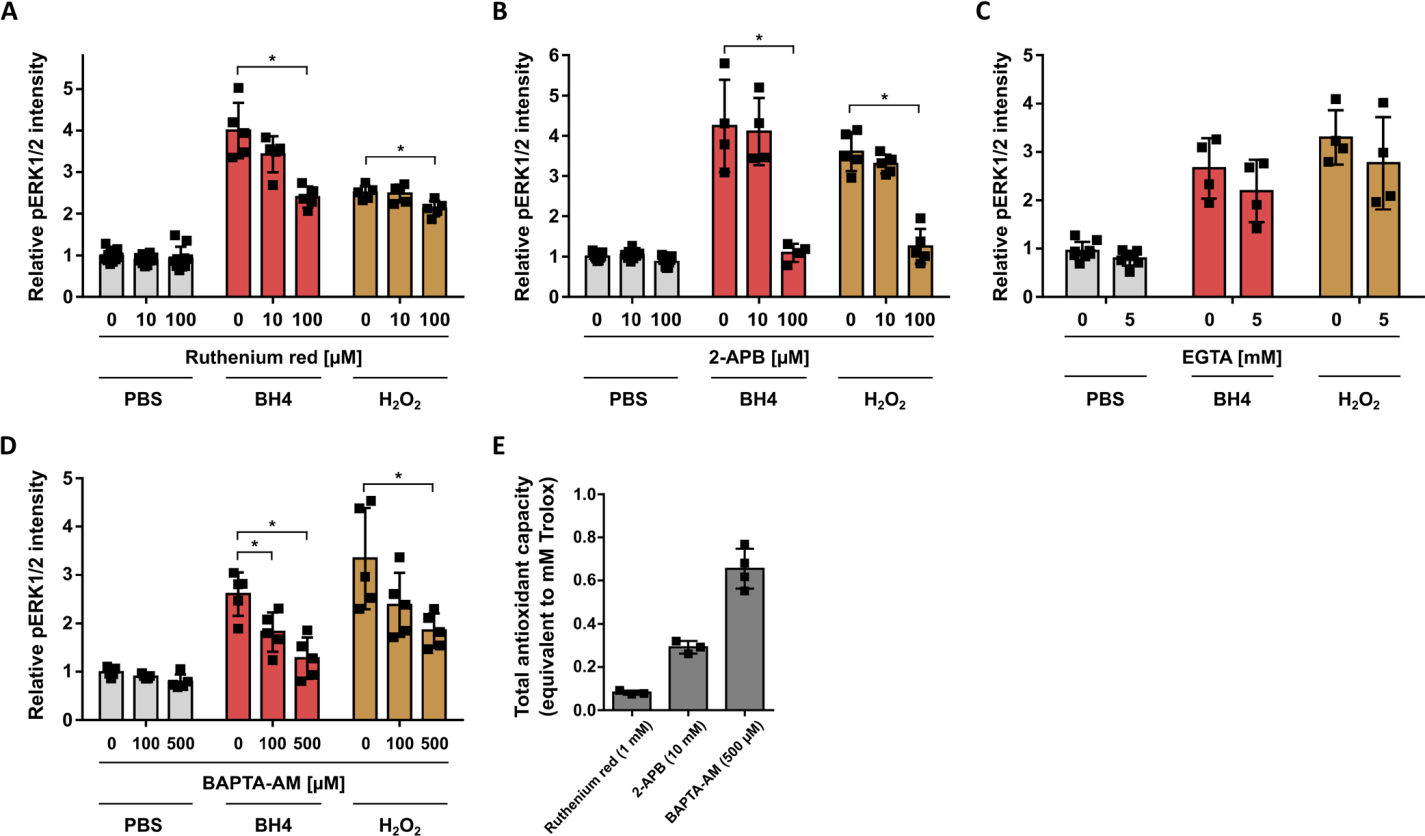
Antioxidizing capacities of ruthenium red, 2‐APB, and BAPTA‐AM interfere with the analysis of a potential Ca^2+^‐dependency of BH4‐ or H_2_O_2_‐induced ERK1/2 signaling in rat dorsal root ganglion neurons. (A–D) Relative pERK1/2 intensity in ex vivo cultured rat DRG neurons (UCHL1^+^) exposed to BH4 (100 μM) or H_2_O_2_ (25 μM) after treatment with the indicated compounds. Statistical significance tested by one‐way ANOVA with Bonferroni multiple comparison testing (A, B, D) or by unpaired, two‐sided *t‐*test (C), in comparison to 0 μM compound (*p < 0.05). Data presented as individual data points representing biological replicates and mean ± SD. Experiments have been performed with independent DRG preparations from *n* = 4–5 animals depicted by individual data points. (E) Colorimetric analysis of total antioxidant capacity of the indicated reagents in PBS using the Total Antioxidant Capacity Colorimetric Assay Kit, following manufacturer's instructions with independent Trolox standard curves (0–1.43 mM) for each replicate. Data presented as individual data points representing biological replicates and mean ± SD. (A–E) Data presented as individual data points representing biological replicates and mean ± SD (A: BH4, *F*(2, 9) = 14.08, *p* = 0.0007; H_2_O_2_, *F*(2, 9) = 6.612, *p* = 0.0116; B: BH4, *F*(2, 9) = 18.37, *p* = 0.0007; H_2_O_2_, *F*(2, 9) = 50.90, *p* < 0.0001; D: BH4, *F*(2, 9) = 12.07, *p* = 0.0013; H_2_O_2_, *F*(2, 12) = 5.161, *p* = 0.0241).

First, we applied the broad‐spectrum Ca^2+^ channel blockers ruthenium red and 2‐APB before exposing rat DRG neurons to BH4 or H_2_O_2_. Both compounds significantly reduced BH4‐ and H_2_O_2_‐induced pERK1/2 levels (Figure 5A,B). However, chelating extracellular Ca^2+^ with EGTA did not reduce pERK1/2 levels upon BH4 or H_2_O_2_ exposure (Figure 5C). Thus, we obtained contradictory results based on whether we interfered with Ca^2+^ signaling using Ca^2+^ channel blockers (ruthenium red, 2‐ABP) or by blocking Ca^2+^ flux by chelating extracellular Ca^2+^ (EGTA). Since EGTA blocks only Ca^2+^ flux through the cell membrane, but ruthenium red and 2‐ABP also block Ca^2+^ flux across intracellular membranes, we hypothesized that intracellular Ca^2+^ flux may drive BH4‐ and H_2_O_2_‐induced pERK1/2 levels. To confirm this hypothesis, we tested whether chelating intracellular Ca^2+^ with BAPTA‐AM would interfere with increased pERK1/2 levels. BAPTA‐AM crosses the cell membrane and selectively chelates Ca^2+^ only after the acetoxymethyl group is removed by cytoplasmic esterases. Indeed, pre‐treatment with BAPTA‐AM reduced BH4‐ and H_2_O_2_‐induced pERK1/2 levels (Figure 5D). To confirm this data obtained by chelating Ca^2+^, we applied several inhibitors that selectively block components of intracellular Ca^2+^ signaling (Figure S2A). Intriguingly, neither depleting the Ca^2+^ reservoir of the endoplasmic reticulum with thapsigargin (Figure S2B), blockage of ryanodine receptors with ryanodine, dantrolene, or JTV‐519 (Figure S2C–E), inhibiting IP3 receptors with heparin (Figure S2F), inhibiting TRPM2 with flufenamic acid, or ACAA (Figure S2G,H), nor inhibiting TRPM8 with ACAA, or M‐8B (Figure S2H,I) reduced BH4‐ or H_2_O_2_‐induced pERK1/2 levels in rat DRG neurons. Moreover, interfering with intracellular Ca^2+^ signaling by inhibiting Ca^2+^/calmodulin‐dependent protein kinases CaMKK with STO‐609 (Figure S2J), or CaMK II with KN‐93, or AIP (Figure S2K,L) did not show an effect on BH4‐ or H_2_O_2_‐induced pERK1/2 levels either. Thus, we again could not reliably confirm the assumption that Ca^2+^ signaling is of relevance for pERK1/2 levels upon BH4 or H_2_O_2_ exposure.

Because ruthenium red, 2‐APB, and BAPTA‐AM were the only compounds to reduce BH4‐ and H_2_O_2_‐induced pERK1/2 levels in our screening approach, we postulated that perhaps not their inhibitory effect on Ca^2+^ signaling but rather their antioxidant capacities explain their inhibitory effect on BH4‐ and H_2_O_2_‐induced pERK1/2 levels (Morihara et al. 2017; He et al. 2018; Taskin et al. 2014; Meinicke et al. 1998; Kessel et al. 2005). To corroborate this, we determined total antioxidant capacities of ruthenium red, 2‐APB, and BAPTA‐AM by colorimetric analysis. Dilution of BAPTA‐AM in PBS yields a cloudy solution, which interferes with the analysis. Therefore, BAPTA has been used instead. The performed colorimetric analysis confirmed that ruthenium red, 2‐APB, and BAPTA possess antioxidizing capacities (Figure 5E). This result indicated that perhaps not their interference with intracellular Ca^2+^ but their antioxidizing capacity reduced BH4‐ and H_2_O_2_‐induced pERK1/2 levels. Due to the narrow detection window of the colorimetric analysis, however, higher concentrations of these compounds was used compared to cell culture experiments. Thus, we cannot fully exclude at this point that intracellular Ca^2+^ flux affects pERK1/2 levels in addition to the newly described H_2_O_2_‐dependent mechanism.

### 3.5 BH4 and H_2_O_2_ Induce Elevated pERK1/2 Levels via B‐Raf and MEK1/2 in Rat DRG Neurons

**FIGURE 6:**
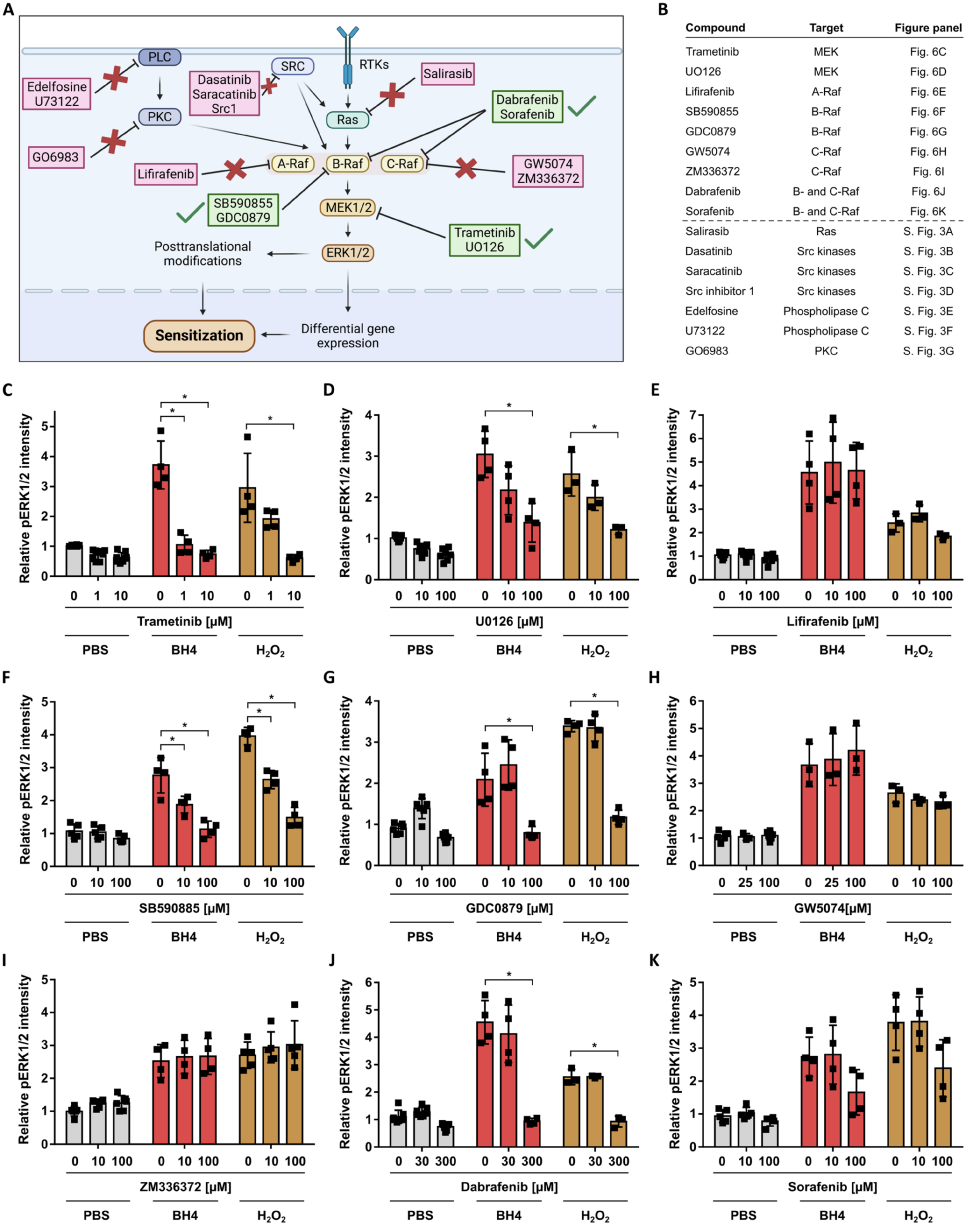
BH4 and H_2_O_2_ induce increased pERK1/2 levels in rat dorsal root ganglion neurons via B‐Raf and MEK. (A) Schematic illustrating relevant components of the ERK1/2 signaling cascade in DRG neurons that were analyzed in this study (PKC, protein kinase C; PLC, phospholipase C; RTKs, receptor tyrosine kinases; SRC, Src kinases). (B) Table listing all compounds used to interfere with ERK1/2 signaling. (C–K) Relative pERK1/2 intensity in ex vivo cultured rat DRG neurons (UCHL1^+^) exposed to BH4 (100 μM) or H_2_O_2_ (25 μM) after treatment with the indicated compounds. Statistical significance tested by one‐way ANOVA with Bonferroni multiple comparison testing, in comparison to 0 μM compound (*p < 0.05). Data presented as individual data points representing biological replicates and mean ± SD. (C: BH4, *F*(2, 9) = 14.08, *p* = 0.0007; H_2_O_2_, *F*(2, 9) = 6.612, *p* = 0.0116; D: BH4, *F*(2, 9) = 9.276, *p* = 0.0065; H_2_O_2_, *F*(2, 6) = 10.60, *p* = 0.0107; F: BH4, *F*(2, 9) = 19.42, *p* = 0.0005; H_2_O_2_, *F*(2, 12) = 75.96, *p* < 0.0001; G: BH4, *F*(2, 9) = 11.19, *p* = 0.0036; H_2_O_2_, *F*(2, 9) = 126.8, *p* < 0.0001; J: BH4, *F*(2, 9) = 26.96, *p* = 0.0002; H_2_O_2_, *F*(2, 6) = 65.74, *p* < 0.0001). Experiments have been performed with independent DRG preparations from *n* = 3–4 animals, depicted by individual data points.

Based on BH4's role as an essential cofactor for important physiological processes, reducing BH4 levels as a therapeutic approach to alleviate chronic pain may lead to undesired neurological and cardiovascular side effects. Therefore, exploring downstream pathways of BH4 can potentially reveal targets to reduce BH4‐induced hypersensitivity while lowering the risk for side effects. After demonstrating that exposing rat DRG neurons to BH4 or its oxidation product H_2_O_2_ induces increased pERK1/2 levels, which is known to induce nociceptor sensitization, we now explored which upstream mediators of ERK1/2 activation are involved (Figure 6A,B).

Activation of MEK1/2 is directly upstream of ERK1/2 (Figure 6A). Thus, we first inhibited MEK1/2 activity in rat DRG neurons with trametinib or U0126 before stimulation with BH4 or H_2_O_2_. As expected, inhibiting MEK1/2 activity significantly reduced BH4‐ and H_2_O_2_‐induced pERK1/2 levels (Figure 6C,D). MEK1/2 can be activated by serine/threonine‐protein kinases of the Raf family, such as A‐Raf, B‐Raf, and C‐Raf (Figure 6A). Inhibition of A‐Raf with Lifirafenib and of C‐Raf with GW5074 or ZM336372 did not significantly reduce BH4‐ or H_2_O_2_‐induced pERK1/2 levels (Figure 6E,H,I). In contrast, inhibiting B‐Raf with the selective inhibitors SB590855 or GDC0879, as well as with the B‐ and C‐Raf dual inhibitor Dabrafenib, led to a significant reduction in pERK1/2 levels upon BH4‐ and H_2_O_2_ exposure (Figure 6F,G,J). In addition, inhibiting B‐Raf with the B‐ and C‐Raf dual inhibitor Sorafenib showed a trend toward reduced pERK1/2 levels (Figure 6K). Based on these experiments, we concluded that BH4 and H_2_O_2_ induce increased pERK1/2 levels via B‐Raf and MEK in rat DRG neurons.

To identify additional components of the BH4‐ and H_2_O_2_‐induced signaling cascade that results in increased pERK1/2 levels, we next inhibited selected modulators of ERK1/2 signaling, such as Ras, Src kinases, phospholipase C, and protein kinase C (Figure 6A,B). However, we did not observe any inhibitory effect on BH4‐ or H_2_O_2_‐induced pERK1/2 levels by inhibiting Ras (Figure S3A), Src kinases (Figure S3B–D), phospholipase C (Figure S3E,F), or protein kinase C (Figure S3G). Thus, while we demonstrate that B‐Raf and MEK1/2 are crucial for BH4‐ and H_2_O_2_‐induced ERK1/2 signaling in rat DRG neurons, our experiments did not identify additional components of this pathway.

## 4 Discussion

Although BH4 has been proposed as a potential pain therapeutic target, BH4‐induced effects on cellular pathways involved in long‐term sensitization, such as activation of ERK1/2, have remained largely unknown (Latremoliere and Costigan 2011; Latremoliere et al. 2015; Tegeder et al. 2006). Our study demonstrates that elevated BH4 levels induce increased pERK1/2 levels in rat DRG neurons via oxidation‐derived H_2_O_2_ and the MAPK signaling cascade components B‐Raf and MEK1/2. These results contribute to our understanding of BH4‐induced hypersensitivity by providing the first evidence for BH4‐induced effects on intracellular signaling with known long‐term sensitizing effects.

In the first experiments, we found that BH4 dose‐dependently induces increased pERK1/2 levels in rat DRG neurons, which is considered a key event in nociceptor sensitization (Andres et al. 2013; Obata and Noguchi 2004). Genetic variants of *GCH1* that show reduced expression of the BH4‐producing enzyme GTP cyclohydrolase 1 in response to activating stimuli are associated with lower pain scores following surgical diskectomy for persistent lumbar root pain (Tegeder et al. 2006) and with delayed cancer pain (Lötsch et al. 2010). Based on our results, it may be postulated that reduced *GCH1* expression results in lower BH4 levels, leading to lower ERK1/2‐dependent sensitization. This may explain the reported lower pain scores in individuals carrying the described *GCH1* variants (Lötsch et al. 2010; Tegeder et al. 2006).

Interestingly, we observed that pERK1/2 levels increase for about 30 min upon BH4 exposure and then recover within the next 1.5 h. Here, the question is whether this recovery is a cellular adaptation to increased BH4 levels, which would indicate that BH4‐induced pERK1/2 may have only short‐term consequences on pERK1/2 levels in vivo, or whether this observation is due to the single administration of BH4 in our in vitro setting, together with its rapid oxidation. Based on the work by others (Heller et al. 2001), as well as our subsequent experiments showing that BH4 is rapidly oxidized in aqueous solutions, we suggest that this recovery to baseline pERK1/2 is due to the rapid oxidation of BH4 and the short half‐life of the produced ROS, not due to cellular adaptations. Since elevated BH4 levels in vivo, which are associated with various pain conditions, are linked to increased expression of BH4 synthesizing enzymes, it may be postulated that BH4 levels are constantly increased (Tegeder et al. 2006). This way, sensory neurons are exposed to increased BH4 levels over longer time periods and may point towards the BH4‐pERK1/2 axis as one BH4‐induced sensitizing pathway in vivo.

During our single‐cell‐based analysis, both nociceptive (RIIβ^+^) and non‐nociceptive (RIIβ^−^) neurons showed increased pERK1/2 levels upon BH4 exposure. Since non‐nociceptive neurons can contribute to pain perception, it will be an important next step to analyze the role of the different neuronal subpopulations in BH4‐associated pain (Tashima et al. 2018).

We then observed that not BH4 itself but rather the production of ROS through its oxidation drives ERK1/2 signaling in rat DRG neurons, which can be modeled by exposing cells to H_2_O_2_. ROS are key players in the pathogenesis of inflammatory and infectious diseases that are sometimes accompanied by still enigmatic pain features (Ostermann and Evering 2024; Alfadda and Sallam 2012; Forman and Zhang 2021; Ostermann et al. 2025). Primary sensory neuron sensitization by ROS‐induced ERK1/2 activity is possibly one underlying mechanism of how these pain features emerge.

Although BH4 oxidation has been shown to produce H_2_O_2_ and O_2_^−^, we found that the elevation of pERK1/2 levels depends only on H_2_O_2_. Interestingly, Forman et al. suggested that among ROS, only H_2_O_2_ fulfills the criteria of a *bona fide* second messenger, while also referring to H_2_O_2_'s capacity to mimic the effect of growth factors when added to cells (Forman et al. 2010). Our results seem to support that specifically H_2_O_2_ and not O_2_^−^ induces ERK1/2 activity, substantiating its role as a second messenger.

As part of this analysis, we also observed that catalase treatment completely abolishes BH4‐induced ERK1/2 activity. Catalase does not cross the cell membrane when added to the cell culture medium. Hence, BH4 is likely oxidized nonenzymatically in the extracellular milieu, and the produced H_2_O_2_ then either crosses the cell membrane or acts on extracellular signaling proteins. DRGs contain various cell types, including fibroblasts, endothelial cells, pericytes, satellite glial cells, Schwann cells, and immune cells such as macrophages and T cells (Techameena et al. 2024; Bhuiyan et al. 2024). We believe it is possible that BH4 oxidation‐derived ROS in the context of increased BH4 secretion during pain conditions may therefore affect other cell types in addition to sensory neurons. In how far this contributes to sensory neuron sensitization needs to be addressed in the following studies.

BH4 has been previously shown to activate a subset of DRG neurons, which was at least partly dependent on BH4‐induced NOS activity, leading to TRPV1 and TRPA1 activation (Miyamoto et al. 2009). Hence, the authors identified a first BH4 downstream mechanism possibly contributing to BH4‐associated hypersensitivity. However, in our study, inhibition of NOS by three different inhibitors did not affect BH4‐induced pERK1/2 levels in rat DRG neurons. Therefore, BH4‐induced TRPV1/TRPA1 activation via increased NOS activity and BH4‐induced pERK1/2 levels potentially occur in parallel, both driving sensory neuron sensitization.

Lastly, we identified the MAPK signaling cascade components MEK1/2 and B‐Raf as upstream mediators of BH4‐ and H_2_O_2_‐induced pERK1/2 levels, which can therefore be blocked by administration of respective inhibitors. It is known that signaling molecules are regulated through redox mechanisms by ROS (Giorgi et al. 2010; Forman et al. 2010; Evans et al. 1997). Thus, our study provides promising new targets to interfere with BH4‐induced hypersensitivity. Notably, the here applied MEK1/2 inhibitor trametinib and B‐Raf inhibitor dabrafenib have been recently granted FDA Accelerated Approval for the treatment of unresectable or metastatic *BRAF* ^*V600E*^‐mutant solid tumors, and novel B‐Raf inhibitors are currently being tested (Hanrahan et al. 2024). Testing these compounds in animal models of BH4‐induced hypersensitivity will therefore be an important next step to explore the analgesic potential of interfering with BH4‐ and ROS‐induced ERK1/2 signaling in primary sensory neurons.

Regarding potential limitations of our study, we focused on pERK1/2 levels as a cellular correlate of sensory neuron sensitization in dissociated DRG neurons in vitro. pERK1/2 levels are a well‐established correlate of ERK1/2 signaling activity and sensory neuron sensitization in vitro and in vivo. Nevertheless, the relevance of the here‐identified mechanism of pERK1/2 induction by BH4 oxidation‐derived H_2_O_2_ for pain development must be validated in subsequent in vitro studies using additional analytical approaches, such as electrophysiological analyses, using human‐derived cells, and must be confirmed in vivo pain models. Our study design relied on pharmacological inhibition to elucidate the mechanism underlying BH4‐induced pERK1/2 levels. This carries the risk that compound‐specific off‐target effects influence experimental outcomes. To minimize the risk that these off‐target effects hamper our interpretations, we ensured to apply more than one compound to inhibit relevant pathways where possible. Nevertheless, this approach cannot control for every potential off‐target effect.

We have demonstrated that exposing rat DRG neurons to BH4 increases pERK1/2 levels via oxidation‐derived H_2_O_2_ and the B‐Raf‐MEK1/2‐ERK1/2 axis. Therefore, elevated BH4 levels as observed in various pain conditions may drive sensory neuron sensitization by activation of MAPK signaling, which could be targeted to attenuate BH4‐induced pain hypersensitivity without the necessity to reduce BH4 levels.

## Author Contributions

**Milad Mohammadi:** conceptualization, methodology, investigation, visualization, writing – review and editing, writing – original draft. **Maike Siobal:** methodology, investigation, writing – review and editing. **Jörg Isensee:** investigation, methodology, writing – review and editing, visualization. **Philipp N. Ostermann:** investigation, visualization, writing – review and editing, writing – original draft. **Tim Hucho:** conceptualization, investigation, writing – review and editing, funding acquisition, project administration.

## Conflicts of Interest

The authors declare no conflicts of interest.

## Peer Review

The peer review history for this article is available at https://www.webofscience.com/api/gateway/wos/peer‐review/10.1111/jnc.70271.

## Supporting information


**Figure S1:** Exposure to TRPA1 agonist AITC and TRPV1 agonist capsaicin induces increased pERK1/2 levels in rat dorsal root ganglion neurons.
**Figure S2:** No effect of the tested Ca^2+^ signaling inhibitors on BH4‐ or H_2_O_2_‐induced pERK1/2 levels in rat dorsal root ganglion neurons.
**Figure S3:** Inhibition of Ras, Src kinases, phospholipase C, or protein kinase C does not affect BH4‐ or H_2_O_2_‐induced pERK1/2 levels in rat dorsal root ganglion neurons.

**Table S1:** Full statistical reports generated by the performed statistical analyses throughout the manuscript.

## Data Availability

The raw data that underlie each figure and support the findings of this study are available from the corresponding authors upon reasonable request.
