# Supplementary material for: BH4 Oxidation‐Derived H2O2 Activates ERK1/2 Signaling via B‐Raf in Rat Dorsal Root Ganglion Neurons
Source: J Neurochem. 2025 Nov 3;169(11):e70271. doi: 10.1111/jnc.70271 (PMC12580945; doi:10.1111/jnc.70271)
Supplement: Supplementary file 1 — Figure S1: Exposure to TRPA1 agonist AITC and TRPV1 agonist capsaicin induces increased pERK1/2 levels in rat dorsal root ganglion neurons. Figure S2: No effect of the tested Ca2+ signaling inhibitors on BH4‐ or H2O2‐induced pERK1/2 levels in rat dorsal root ganglion neurons. Figure S3: Inhibition of Ras, Src kinases, phospholipase C, or protein kinase C does not affect BH4‐ or H2O2‐induced pERK1/2 levels in rat dorsal root ganglion neurons. [file JNC-169-0-s001.pdf]

BH4 oxidization-derived H_2O_2 activates ERK1/2 signaling via B-Raf in rat dorsal root ganglion neurons

Milad Mohammadi, Maike Siobal, Jörg Isensee, Philipp N. Ostermann[#*], Tim Hucho[#*]

Translational Pain Research, Department of Anesthesiology and Intensive Care Medicine, Faculty of Medicine and University Hospital Cologne, University of Cologne, 50931 Cologne, Germany

[#]shared last-authors

[*]Correspondence: P.N.O.: e-mail: philipp.ostermann@uk-koeln.de; Tel.: +49 221 478-87591 and T.H.: e-mail: tim.hucho@uk-koeln.de; Tel.: +49 221 478 97760

Supplementary figures:

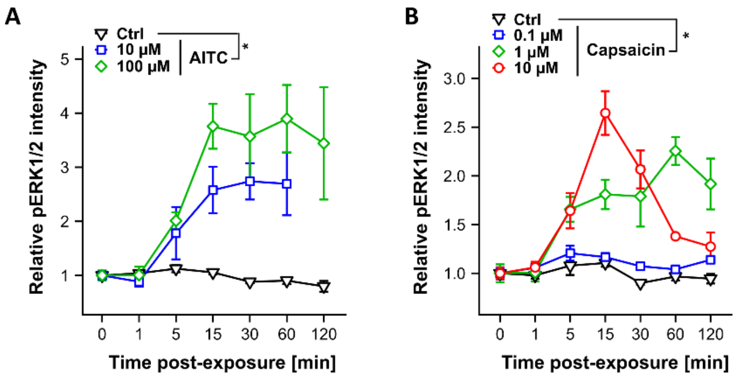

S. Fig. 1 Exposure to TRPA1 agonist AITC and TRPV1 agonist capsaicin induces increased pERK1/2 levels in rat dorsal root ganglion neurons (**A - B**) Relative pERK1/2 intensity in *ex vivo* cultured rat DRG neurons (UCHL1$^+$) exposed to increasing concentrations of AITC (**A**) or capsaicin (**B**) with DMSO as control (Ctrl) for up to 120 minutes. Statistical significance tested between control and grouped compound conditions by two-way ANOVA with Bonferroni's test (*$p < 0.05$; (**A**) $F (2, 63) = 39.221$, $p < 0.001$; (**B**) $F (3, 84) = 61.742$, $p < 0.001$). Data presented as mean ± SD. Experiments have been performed with independent DRG preparations from n = 4 animals.

A

Compound	Ca^{2+}-relevant target	Figure panel
Thapsigargin	Sarcoplasmic/endoplasmic reticulum Ca^{2+}-ATPase	S. Fig. 2B
Ryanodine	Ryanodine receptors (RyR)	S. Fig. 2C
Dantrolene	Ryanodine receptors (RyR)	S. Fig. 2D
JTV-519	Ryanodine receptors (RyR)	S. Fig. 2E
Heparin	IP3 receptors	S. Fig. 2F
Flufenamic acid	TRPM2	S. Fig. 2G
ACAA	TRPM2/TRPM8	S. Fig. 2H
M-8B	TRPM8	S. Fig. 2I
STO-609	CaMKK	S. Fig. 2J
KN-93	CaMK II	S. Fig. 2K
AIP	CaMK II	S. Fig. 2L

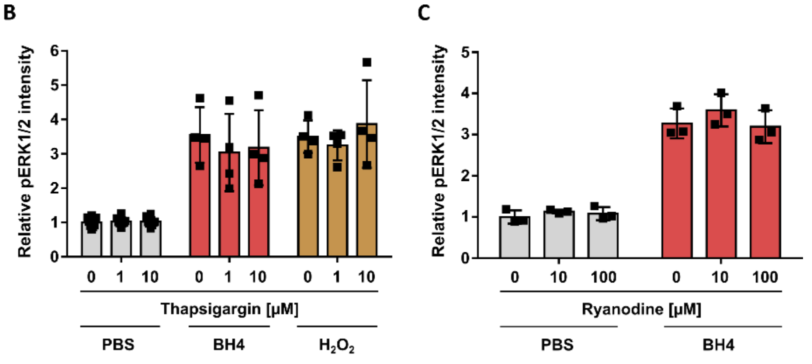

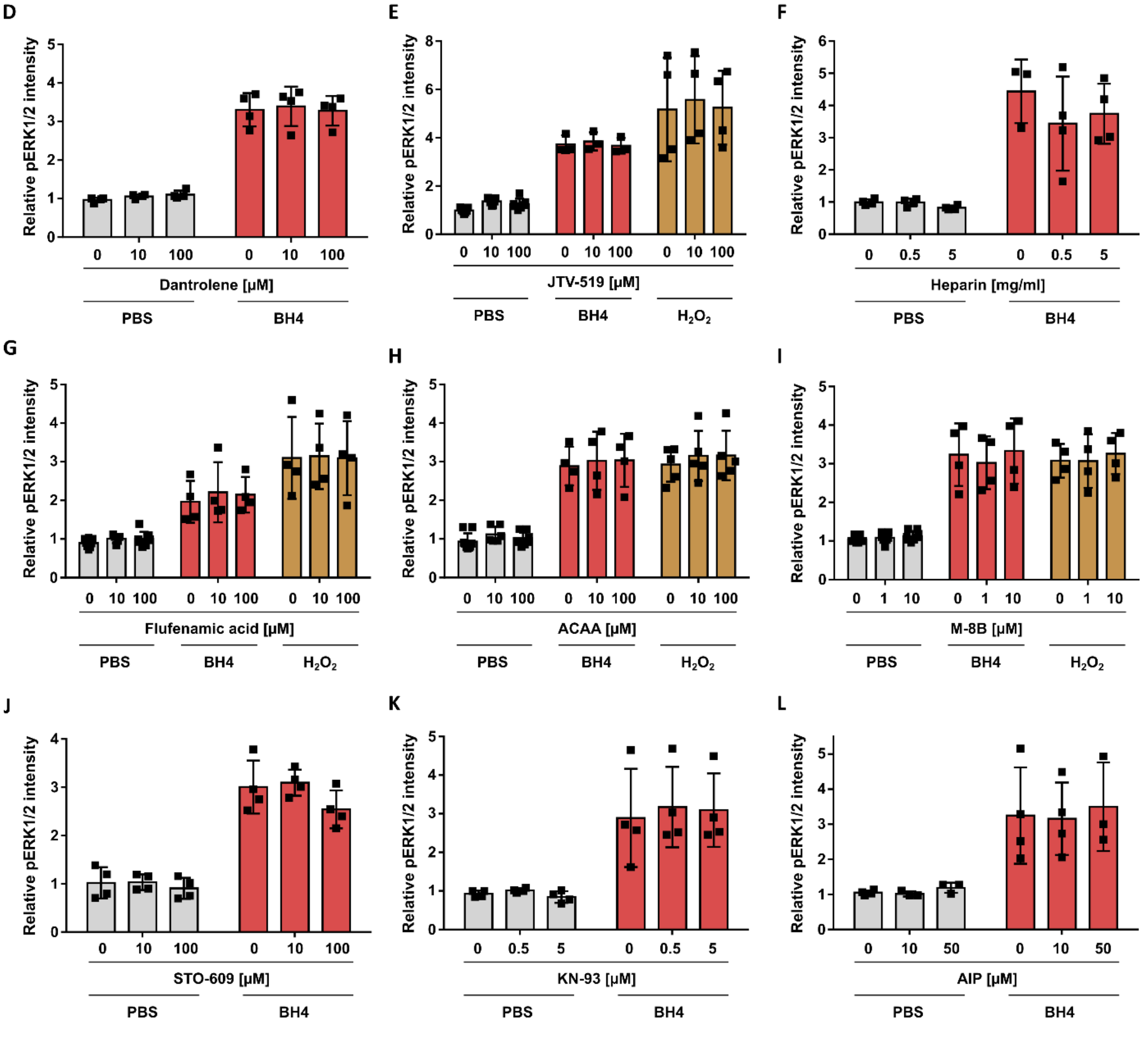

S. Fig. 2 No effect of the tested Ca^{2+} signaling inhibitors on BH4- or H_2O_2-induced pERK1/2 levels in rat dorsal root ganglion neurons. (**A**) Table listing all compounds used to interfere with Ca^{2+} signaling in this figure (**B - L**) Relative pERK1/2 intensity in *ex vivo* cultured rat DRG neurons ($UCHL1^+$) exposed to BH4 (100 µM) or H_2O_2 (25 µM) after treatment with the indicated compounds. Statistical significance tested by one-way ANOVA with Bonferroni multiple comparison testing, in comparison to 0 µM compound in each group (*$p < 0.05$); **B**(BH4): $F(2, 9) = 0.2655$, $p = 0.7726$; **B**(H_2O_2): $F(2, 9) = 0.5745$, $p = 0.5823$; **C**(BH4): $F(2, 6) = 0.8940$, $p = 0.4573$; **D**(BH4): $F(2, 9) = 0.07560$, $p = 0.9278$; **E**(BH4): $F(2, 6) = 0.1937$, $p = 0.8289$; **E**(H_2O_2): $F(2, 9) = 0.05380$, $p = 0.9479$; **F**(BH4): $F(2, 8) = 0.6425$, $p = 0.5511$; **G**(BH4): $F(2, 9) = 0.1758$, $p = 0.8416$; **G**(H_2O_2): $F(2, 9) = 0.003276$, $p = 0.9967$; **H**(BH4): F(2,

9) = 0.06453, p = 0.9379; **H**(H_2O_2): $F(2, 12) = 0.2569$, $p = 0.7776$; **I**(BH4): $F(2, 9) = 0.1590$, $p = 0.8553$; **I**(H_2O_2): $F(2, 9) = 0.1506$, $p = 0.8623$; **J**(BH4): $F(2, 9) = 1.996$, $p = 0.1916$; **K**(BH4): $F(2, 9) = 0.07010$, $p = 0.9328$; **L**(BH4): $F(2, 8) = 0.06896$, $p = 0.9339$. Data presented as individual data points representing biological replicates and mean ± SD. Experiments have been performed with independent DRG preparations from n = 4 - 5 animals depicted by individual data points.

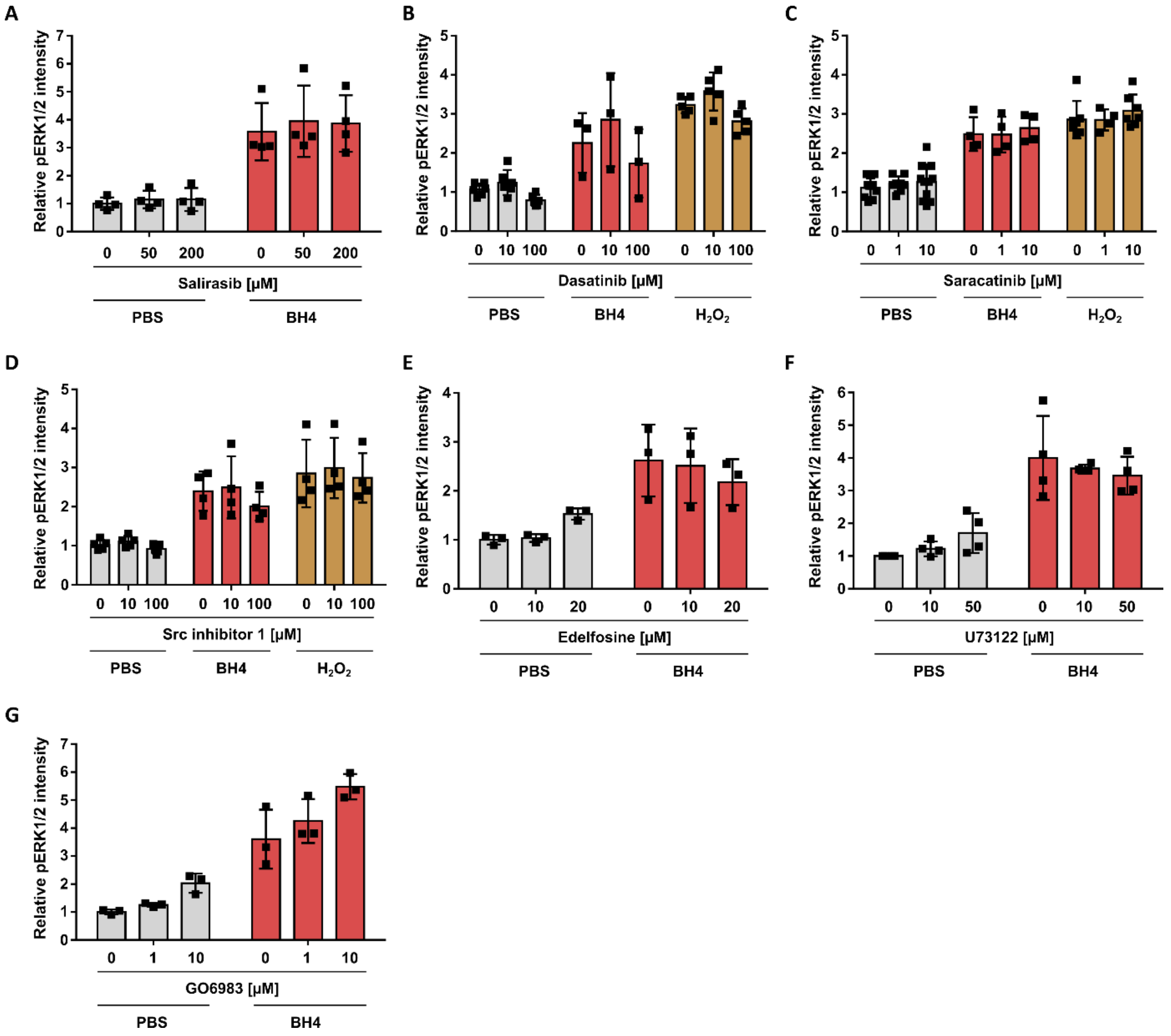

S. Fig. 3 Inhibition of Ras, Src kinases, phospholipase C, or protein kinase C does not affect BH4- or H_2O_2-induced pERK1/2 levels in rat dorsal root ganglion neurons. (**A - G**) Relative pERK1/2 intensity in *ex vivo* cultured rat DRG neurons (UCHL1[+]) exposed to BH4 (100 µM) or H_2O_2 (25 µM) after treatment with the indicated compounds. Statistical significance tested by one-way ANOVA with Bonferroni multiple comparison testing, in comparison to 0 µM compound (*$p < 0.05$). **A**(BH4): $F(2, 9) = 0.1257$, $p = 0.8834$; **B**(BH4): $F(2, 6) = 1.017$, $p = 0.4164$; **B**(H_2O_2): $F(2, 12) = 5.661$, $p = 0.0186$; **C**(BH4): $F(2, 9) = 0.1930$, $p = 0.8278$; **C**(H_2O_2): $F(2, 15) = 0.6549$, $p = 0.5337$; **D**(BH4): $F(2, 9) = 0.7618$, $p = 0.4947$; **D**(H_2O_2): $F(2, 9) = 0.1082$, $p = 0.8986$; **E**(BH4): $F(2, 6) = 0.3587$, $p = 0.7126$; **F**(BH4): $F(2, 9) = 0.4438$, $p = 0.6549$; **G**(BH4): $F(2, 6) = 4.238$, $p = 0.0712$. Data presented as individual data points

representing biological replicates and mean ± SD. Experiments have been performed with independent DRG preparations from n = 3 - 5 animals.
